# Brain areas activated during visual learning in the cichlid fish *Pseudotropheus zebra*

**DOI:** 10.1007/s00429-023-02627-w

**Published:** 2023-03-15

**Authors:** R. Calvo, M. H. Hofmann, V. Schluessel

**Affiliations:** Institute of Zoology, Rheinische Friedrich-Wilhelms-Universität Bonn, Poppelsdorfer Schloss, Meckenheimer Allee 169, 53115 Bonn, Germany

**Keywords:** Cichlid, pS6, Brain, Visual learning, Behavior, Neural activity

## Abstract

The neural correlates of most cognitive functions in fish are unknown. This project aimed to identify brain regions involved in visual learning in the cichlid fish *Pseudotropheus zebra*. The expression of the protein pS6 was measured in 19 brain areas and compared between groups of individuals subjected to four different behavioral contexts (control, avoidance, trained, and novelty groups). Control group individuals were sacrificed with minimal interactions. Fish in the avoidance group were chased with a net for an hour, after which they were sacrificed. Individuals in the trained group received daily training sessions to associate a visual object with a food reward. They were sacrificed the day they reached learning criterion. Fish in the novelty group were habituated to one set of visual stimuli, then faced a change in stimulus type (novelty stimulus) before they were sacrificed. Fish in the three treatment groups showed the largest activation of pS6 in the inferior lobes and the tectum opticum compared to the control group. The avoidance group showed additional activation in the preoptic area, several telencephalic regions, the torus semicircularis, and the reticular formation. The trained group that received a food reward, showed additional activation of the torus lateralis, a tertiary gustatory center. The only area that showed strong activation in all three treatment groups was the nucleus diffusus situated within the inferior lobe. The inferior lobe receives prominent visual input from the tectum via the nucleus glomerulosus but so far, nothing is known about the functional details of this pathway. Our study showed for the first time that the inferior lobes play an important role in visual learning and object recognition.

**Supplementary Information:**

The online version contains supplementary material available at 10.1007/s00429-023-02627-w.

## Introduction

Fish have been the subject of a large number of visual discrimination experiments, which have shown that fish possess many of the same cognitive abilities as birds and mammals (for reviews see Brown et al. 2011; Schluessel 2015). Such experiments range from object recognition and categorization (e.g. Neri 2012; Schluessel and Bleckmann 2012), recognition of symmetrical symbols (Schluessel et al. 2014), size, shapes, and form constancy (Douglas et al. 1988; Schuster et al. 2004; Frech et al. 2012; Schluessel et al. 2014; DeLong et al. 2018), to numerical competency (e.g. Agrillo et al. 2017; Mehlis et al. 2015; Schluessel et al. 2022) as well as optical illusions (e.g., Wyzisk and Neumeyer 2007; Fuss et al. 2014; Agrillo et al. 2020). Unfortunately, only a few studies so far have described where and how cognitive information is processed in the fish brain. With the exception of the lateral and medial division of the dorsal telencephalon, which are supposed to be homologs of the hippocampus and the pallial amygdala of land vertebrates, respectively, the neural correlates for most cognitive functions in fish are still unknown (for reviews see Rodríguez et al. 2006; Broglio et al. 2011; Ebbesson and Braithwaite 2012; Kotrschal et al. 2014; Calvo and Schluessel 2021). This includes functions and cognitive involvement of other areas within the telencephalon, as well as the diencephalon and regions outside the forebrain. The diencephalon includes the habenula, the thalamus, the hypothalamus, and the posterior tubercular region, which is highly derived in many teleost groups (Ahrens and Wullimann 2002). In addition, teleosts have a prominent visual pathway extending from the tectum over the nucleus corticalis and the nucleus glomerulosus to the inferior lobes (Wullimann and Meyer 1990; Butler et al. 1991; Shimizu et al. 1999; Ahrens and Wullimann 2002; Yang et al. 2007). Other parts of the inferior lobes and the corpus mammilaris project back to the tectum (Hagio et al. 2018; Sawai et al. 2000). In addition, the corpus mammilare and the commissural preglomerular nucleus have extensive projections to the dorsal telencephalon (Murakami et al. 1983; Sawai et al. 2000). This shows that a large part of visual information processing and integration with other senses is taking place in the posterior tubercle/inferior lobe region in teleosts. However, these regions have not been included in studies that try to localize cognitive functions in fishes.

Markers for immediate early gene (IEG) expression have been used during the last two decades to identify brain areas involved in different cognitive functions in fishes. C-fos and egr-1 are generally the two main proteins assessed, which can be detected by immunocytochemistry inside the nucleus of activated neurons. However, the availability of antibodies for fishes is problematic and many studies used in situ hybridization and PCR on micro dissected brain parts to localize the expression of genes correlated with the activation of neurons. More recently, the phosphorylated ribosome marker pS6 has become a popular alternative to visualize neural activation in fish (e.g. Benítez-Santana et al. 2017; Travanca dos Santos 2017; Butler et al. 2018; Fischer et al. 2018; York et al. 2018; Montesano et al. 2019; Tripp et al. 2019; York et al. 2019; Baran and Streelman 2020; Butler et al. 2020; Maruska et al. 2020; Tripp et al. 2020; Chen et al. 2021; Dunlap et al. 2021; Nunes et al. 2021; Schuppe et al. 2021; Suzuki et al. 2021; Scaia et al. 2022). S6 protein is a component of the 40S ribosomal subunit. Its inducible phosphorylation, which occurs in response to a large variety of stimuli, was the first post-translational modification described in ribosomal proteins (Gressner and Wool 1974; Meyuhas 2008) and has attracted attention since its discovery in 1974 in rat liver regeneration (Gressner and Wool 1974). The phosphorylation of S6 is supposed to be a more sensitive method than IEG markers since it does not require gene activation and antibodies are readily available due to the highly conserved S6 protein sequence.

To investigate brain areas involved in different visual learning tasks, we used pS6 antibodies in the cichlid *Pseudotropheus zebra*. To facilitate the measurement of as many brain areas as possible, we developed an automated image analysis procedure and analyzed 19 brain areas in 40 individuals from four groups yielding more than 3000 individual areas extracted from the stained image stacks. The activation of these areas ranging from sensory to motor centers was then compared with three different learning situations that each involved different sensory, cognitive, and locomotor components.

## Materials and methods

### Animals

Animals used in this study (*n* = 40) belong to the species *Pseudotropheus zebra,* also known as *Zebra mbuna* (Konings and Stauffer 1997), *Metriaclima zebra* or *Maylandia zebra* (Boulenger 1899). This species belongs to the family Cichlidae (www.fishbase.org). Cichlids are teleosts and represent one of the most varied extant vertebrate radiations (Seehausen 2006), showing a high degree of variability in terms of trophic morphology, (including specialist algal scrapers, planktivorous, insectivores, piscivores, paedophages, snail crushers, and fin biters (Albertson et al. 1999), color pattern (Konings 1995; Albertson et al. 1999), as well as polyandrous mating systems (Kellogg et al. 1995; Albertson et al. 1999). For this reason, African cichlids offer an unequaled system of animals to study cognition, molecular evolution, speciation, and ecological plasticity (van Staaden et al. 1995; Salzburger et al. 2005).

### Experimental procedures—behavioral experiments

Each fish was kept individually, without any social contact, in a tank that served both as a holding as well as an experimental tank. All fish were kept in isolation for at least one week prior to being sacrificed (control group) or to being used in behavioral experiments (avoidance, trained and novelty groups). The isolation was necessary to avoid the activation of brain areas involved in social interactions. Walls and floor of each tank consisted of light grey PVC, while the front was made of white frosted plexiglass. A grey partition was inserted into the middle of the tank separating a back from a front compartment; the partition was fitted with a passage for the fish, that could be closed with the help of a transparent guillotine door during the training of the fish. In the back compartment of the tank, a fish shelter, a heating element, a filter system as well as a pump were placed. The water temperature was kept at about 25 °C.

**Fig. 1:**
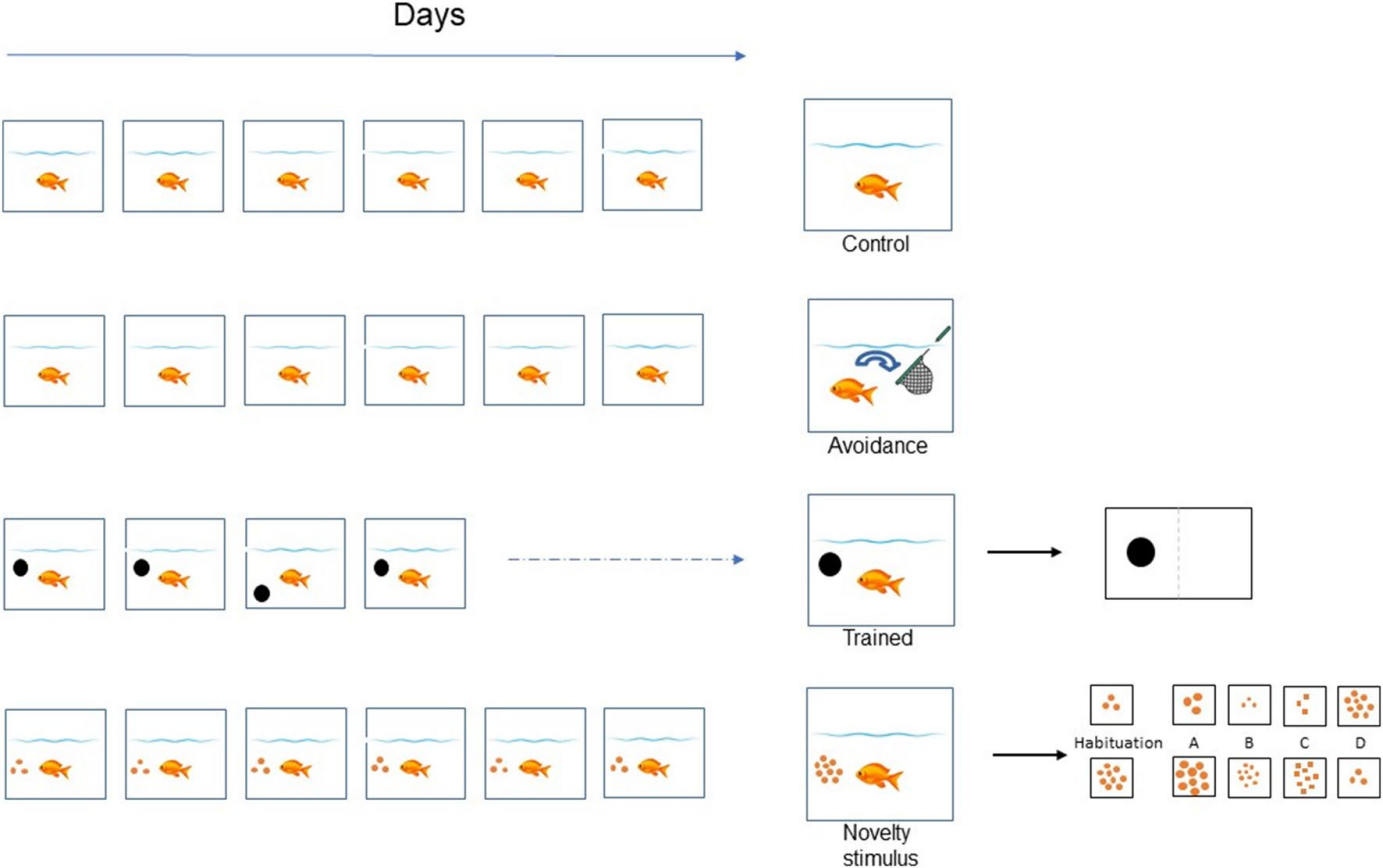
Schematic representation of the four different experimental groups, highlighting the different learning conditions. For trained and novelty stimulus groups, relevant stimuli are presented on the right

Figure 1 shows a schematic representation of the four different experimental groups. For the trained and the novelty stimulus group the stimuli presented during the behavioral experiments are shown on the right-hand side of the figure.

#### Control group

Fish in the control group (*N* = 10) did not receive any treatment. They were fed once daily, except on the day of sacrifice to prevent any activation of sensory brain areas involved in olfactory and gustatory pathways. After being kept in isolation without any social contact for seven days, fish were taken out of the tank and sacrificed.

#### Avoidance group

Fish in the avoidance group (*N* = 10), were fed once daily, except on the day of the behavioral procedure. After being kept for seven days in isolation and immediately prior to the behavioral procedure, they were moved to a new compartment. Fish were then chased with a net for one hour before being sacrificed 90 min after the chase ended.

#### Trained group

Fish in the trained group (*N* = 10) were kept in isolation for one week before the training started. On the inside of each plexiglass front wall of the tank, two food holders were installed, consisting of two small pieces of plastic pipe which were attached to the wall with suckers; in each pipe, a hose filled with food was inserted. On the other end, the hose was connected to a syringe through which the food delivery was controlled remotely. With the help of a plastic divider, the front compartment was divided into a right and left compartment. Parallel and 3 cm away from the frontal wall a line indicated the start of the decision-making area into which the fish had to swim in order for its choice to be valid. Please refer to Schluessel et al. (2018) for a picture of the experimental setup.

Before the actual training commenced, the experimentally naive animals were given time to get used to the experimental setup and to feeding from the food holders in the decision area. The guillotine door was open so that the fish could freely swim throughout both compartments of the aquarium. After the fish had learned to swim through the open guillotine door and collect food from the dispenser at the front of the tank while the projector (no stimulus presentation) was on, training started. Experiments were conducted daily, twice a day always at the same time, during daylight hours. At the beginning of the experiment, the two training symbols (a black dot over a white background (positive stimulus) and a white background without a symbol (alternative, negative stimulus), Fig. 1) were projected onto the plexiglass front with the help of an LCD projector (ES 521 Optoma, DLP^®^, China) which was located in front of the tank and connected to a notebook. The stimuli were projected on the right and left side, just below the respective food holders and at the same height as the guillotine door to allow the fish to see them, even from the posterior area of the tank. For each of the ten trials within a session, the position of the positive stimulus was randomly determined prior to the experiments (creating a rotational scheme) during which the positive stimulus was shown five times on each side of the tank in total; however, it was never shown more than twice in a row on the same side. In total, four rotational schemes were used consecutively over every four sessions. Before each trial, the door was closed, restricting the fish to the back compartment. With the help of a webcam, positioned above the experimental tank, it was possible to observe the behavior of the animal in the tank, without affecting its decision. The guillotine door was then remotely opened and the fish allowed to enter the front compartment. Once the door was opened, the fish had to make a choice within 2 min, otherwise the trial was terminated. To prevent olfactory cues, both feeders were baited in each trial and always simultaneously re-baited to prevent unintentional cuing by the experimenter.

Trial time was taken from the time the individual passed through the guillotine door with the tip of its mouth, until crossing the decision line in front of the projector (indicating that the choice was made, see Schluessel et al. 2018 for details).

A correct choice was rewarded with food. Immediately following an incorrect choice or after consuming the food, the fish was ushered back into the posterior part of the tank.

The learning criterion was established to be seven or more correct decisions out of ten trials in three consecutive sessions. During the experiment, the pumps and heating rods in the back compartment of the respective tanks were turned off. Immediately after the learning criterion was achieved, fish went through as many trials as possible for the duration of 1 h, called a “supersession”. The animals were then sacrificed 90 min after the supersession was finished.

#### Novelty stimulus group

Fish in the novelty stimulus group (*N* = 10) were kept in isolation for one week before starting the experiment. For this group, a modification of the habituation-dishabituation experiment elaborated by Messina and colleagues was used (Messina et al. 2022). The setup was the same as for the trained fish. The stimuli used for the habituation and the novel stimuli are shown in Fig. 1. During habituation, one set of either three or nine dots on a white background was projected onto the plexiglass with the LCD projector. After a delay of 30 s, food was released near the stimulus. The stimulus was turned off two min after the food delivery. Five min later, a new trial was started. One session consisted of four trials and three sessions were done each day. On the last day, the fish received only one session. After that, fish were left in their respective tanks for 5 h before one of the novelty stimuli was shown for 30 s (dishabituation phase). Fish were randomly assigned to the five novelty stimuli. The novel stimulus differed from the habituated one in size, shape, or number of dots (see Fig. 1). No food was provided during these test trials. The animals were sacrificed 90 min after the dishabituation phase was terminated.

### Immunohistochemistry

All fish were anesthetized with Tricaine methanesulfonate (MS-222). The brain was removed and fixed overnight in 4% paraformaldehyde (PFA) at 4 °C, then cryoprotected overnight in 30% sucrose at 4 °C. The following day, the brain was embedded in O.C.T. compound (freezing medium, Leica Biosystem Richmond) and frozen at − 20 °C. Thirty- five µm thick sections were cut at − 20 °C with a cryostat (Leica CM1520) and mounted on gelatin-coated slides in three series, then stored until immunohistochemistry (IHC).

To minimize differences in stain intensity, IHC was performed twice within three days, each time including 5 brains for each group (i.e. 5 control, 5 avoidance, 5 trained and 5 novelty).

During the IHC, sections were rehydrated by washes in PBS drops; then a post-fixation procedure was performed with 4% PFA drops for 10 min, followed by several washes in PBS. The sections were incubated for 30 min in distilled water (H_2_O_d_) containing 1.5% H_2_O_2_ to deactivate endogenous peroxidase, followed by several washes in PBS. Slides were then blocked in 10% normal goat serum (NGS) for 1 h. The sections were transferred to a primary pS6-antibody solution (5% NGS / 1X PBS—0.3% Triton X-100, rabbit anti-pS6 (Ser235/236) antibody, Cell Signalling 2211S: 1:1000) overnight at 4 °C, before being washed several times in PBS.

The following day, the second antibody reaction (VECTASTAIN biotinylated anti-rabbit IgG secondary antibody, Vector Labs., USA:1:500) was performed in 5% NGS/1X PBS—0.3% Triton X-100, followed by repeated washes in PBS. Then, signal amplification was initiated using the ABC method (1:1500, 1X PBS—0.3% Triton X-100, VECTASTAIN ABC-Peroxidasekit (PK6100 elite), Vector Labs., USA) for 1 h at RT. Following several rinses in PBS, the antibody-Avidin–Biotin complex was visualized using the chromogen-solution (one 3,3′-Diaminobenzidine-Tetrahydrochloride (DAB) buffer tablet (Merck KGaA, Germany) dissolved in 10 ml H_2_O, 500 μL 1% ammonium nickel sulfate, 12 μL 30% H_2_O_2_) for ~ 15 min, resulting in a deep greyish reaction product confined to the cell bodies of activated neurons. The reaction was stopped by several washes in PBS. Subsequently, sections were dehydrated in ascending alcohols before coverslipping from xylene with Eukitt (Carl Roth, Germany).

For the current study, the specificity of pS6 antibody was checked by replacing either the primary or secondary antibodies with PBS, showing no reaction product. No other test of the specificity of the antibody was performed.

The same pS6 antibody from Cell Signaling has been used successfully in several studies on fish (Beckers et al. 2019, 2021; Montesano et al. 2019; Tripp et al. 2019, 2020; Chen et al. 2021; Dunlap et al. 2021; Nunes et al. 2021; Schuppe et al. 2021; Scaia et al. 2022), including cichlids (Butler et al. 2018, 2019, 2020; Maruska et al. 2020). In cichlid, the antibody has been validated in *Astatotilapia burtoni* (Butler et al. 2020) by western blot which produced a single band at 32 kDa. The same result has been obtained in midshipman (*Porichthys notatus,* Tripp et al. 2019; Schuppe et al. 2021). Furthermore, the antibody detects endogenous levels of ribosomal protein S6 only when phosphorylated at serine 235 and 236, which are among the phosphorylation sites on S6 that are evolutionarily conserved (Meyuhas 2008). In sections, the antibody binds to ribosomes in the endoplasmatic reticulum (Nissl substance of neurons). The nucleus and the dendrites and axons are not stained. This staining pattern was also observed in our material.

### Data analysis

**Fig. 2:**
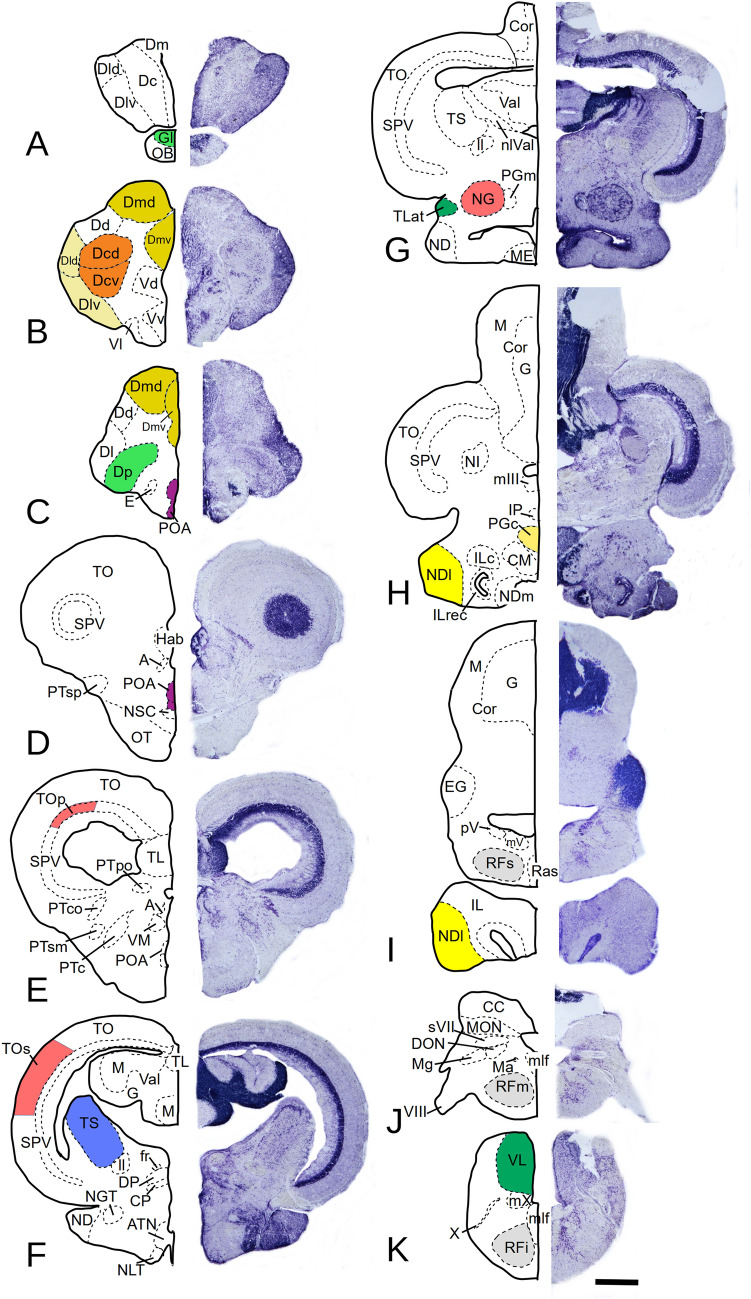
Cryostat sections of the *P. zebra* brain. The right side shows the micro photos of the original sections after Nissl staining, and the left side shows a schematic drawing of identifiable areas and nuclei (see list of abbreviations). Areas analyzed are highlighted in different colors. Scale bar: 500 μm for each section

To measure the activation of pS6, slides were scanned with a custom build scanning stage attached to a Zeiss microscope with a resolution of 1.6 μm. Individual sections were extracted from the scanned slides and an image stack was created for each brain. In these image stacks, the boundaries of different brain areas were marked. Figure 2 shows the location of the different brain areas analyzed, listed in Table 1. Even areas without pS6 stained cells could be identified by a slight unspecific background staining due to the ABC kit. Details about how the areas were identified and other image analysis procedures can be found in the supplementary materials.

**Table 1:** List of brain areas analyzed

**Telencephalon**
*OB* olfactory bulb/granular layer
*Dmd* dorsal subdivision of the medial division of the dorsal telencephalon
*Dmv* ventral subdivision of the medial division of the dorsal telencephalon
*Dldm* dorsal subdivision of the lateral division of the dorsal telencephalon, pars magnocellularis
*Dldp* dorsal subdivision of the lateral division of the dorsal telencephalon, pars parvocellularis
*Dlv* ventral subdivision of the lateral division of the dorsal telencephalon
*Dcd* dorsal subdivision of the central division of the dorsal telencephalon
*Dcv* ventral subdivision of the central division of the dorsal telencephalon
*Dp* posterior division of the dorsal telencephalon
*POA* preoptic area
**Diencephalon**
*NG* nucleus glomerulosus
*NDI* nucleus diffusus lobi inferioris
*PGc* commissural preglomerular nucleus
**Mesencephalon**
*TOp* periventricular layer of the tectum opticum
*TOs* superficial layer of the tectum opticum
*TS* torus semicircularis
*TLat* torus lateralis
**Brainstem**
*RF* reticular formation
*VL* vagal lobe

The segmentation of the areas was the only step with user intervention and was performed blind, i.e. the user selected an area to be marked and the computer presented an image stack randomly from the four groups without showing any labels that could identify the group. The user then had to identify the area of interest in the image stack and define an area for the subsequent analysis. The user was also instructed to exclude possible artefacts due to the cutting and staining procedures. For a certain brain part, several areas were segmented from both sides of the brain or from different sections. This resulted in more than 3000 individual areas defined for the 19 brain regions in the 40 animals belonging to the four groups. An automated image analysis workflow was used to measure the segmented areas. The details are described in the Supplementary methods. The results of these measurements are shown in Fig. 4, ‘Group means’. Each value is the ratio of the area stained by the pS6 antibody divided by the total area segmented for each brain part, averaged for each group.

Next, a *U*-test (alglib software package) was performed for each brain part comparing the learning groups with the control group to check for significant differences (Fig. 4, *U*-Test Group/Control). Lastly, the activation in each brain area was calculated by dividing the pS6 stain level into the three learning groups by the staining level in the control group and log-transformed (Fig. 4 Relative log(Group/Control)).

## Results

Behavioral situations require input from different sets of sensory, cognitive, and motor components. To correlate the activation of different brain parts with the four experimental groups, the behavioral components that characterized the last hour before the fish were sacrificed were analyzed. Table 2 lists the four experimental groups and the components that may play a role for the brain areas activation pattern.

**Table 2:** List of behavioral components that may correlate with the brain areas activation pattern

	**Olfactory**	**Spatial cognition**	**Stress**	**Vision**	**LL/auditory**	**Taste**	**Locomotion**
Control	–	–	–	–	–	–	–
Avoidance	+ + + +	+	+ + + +	+ + + +	+ +	–	+ + + +
Trained	+	+ +	+	+ + + +	+	+ + +	+ + +
Novelty	–	–	–	+ + + +	–	–	+

In the control group, no specific or intentional behavior was elicited before the fish were taken out of the tank and sacrificed (Table 2, ‘Control’). Individuals in the avoidance group were transferred to a new environment and chased with a net. These two modifications introduced different sets of behavioral responses (Table 2, ‘Avoidance’). First of all, the presence of a net chasing the fish created a stressful situation; additionally, there were visual/auditory/hydrodynamic stimuli due to the movement of the net. Furthermore, new olfactory, as well as new spatial cues were introduced due to the presence of the new environment. Lastly, the forced movement caused by the chasing treatment induced a strong motor component.

In the trained group, the primary stimulus was a visual target, but auditory and hydrodynamic inputs cannot be completely excluded due to handling procedures (Table 2, ‘Trained’). Since the fish were rewarded with food, gustatory and olfactory stimulation was also present. The training itself involved locomotion and possibly a small spatial component because the fish had to pass the guillotine door and to swim to the target to get a reward.

The novelty group was more comparable to the control group than the avoidance or the trained groups. No sensory stimulation was introduced other than the visual image. Fish were not actively trained and did not receive food before being sacrificed hence avoiding the presence of gustatory-olfactory stimulation. Furthermore, the fish did not enter a new environment, thus limiting the introduction of a spatial component. The only new stimulus introduced on the last day was the visual stimulus (Table 2, ‘Novelty’).

**Fig. 3:**
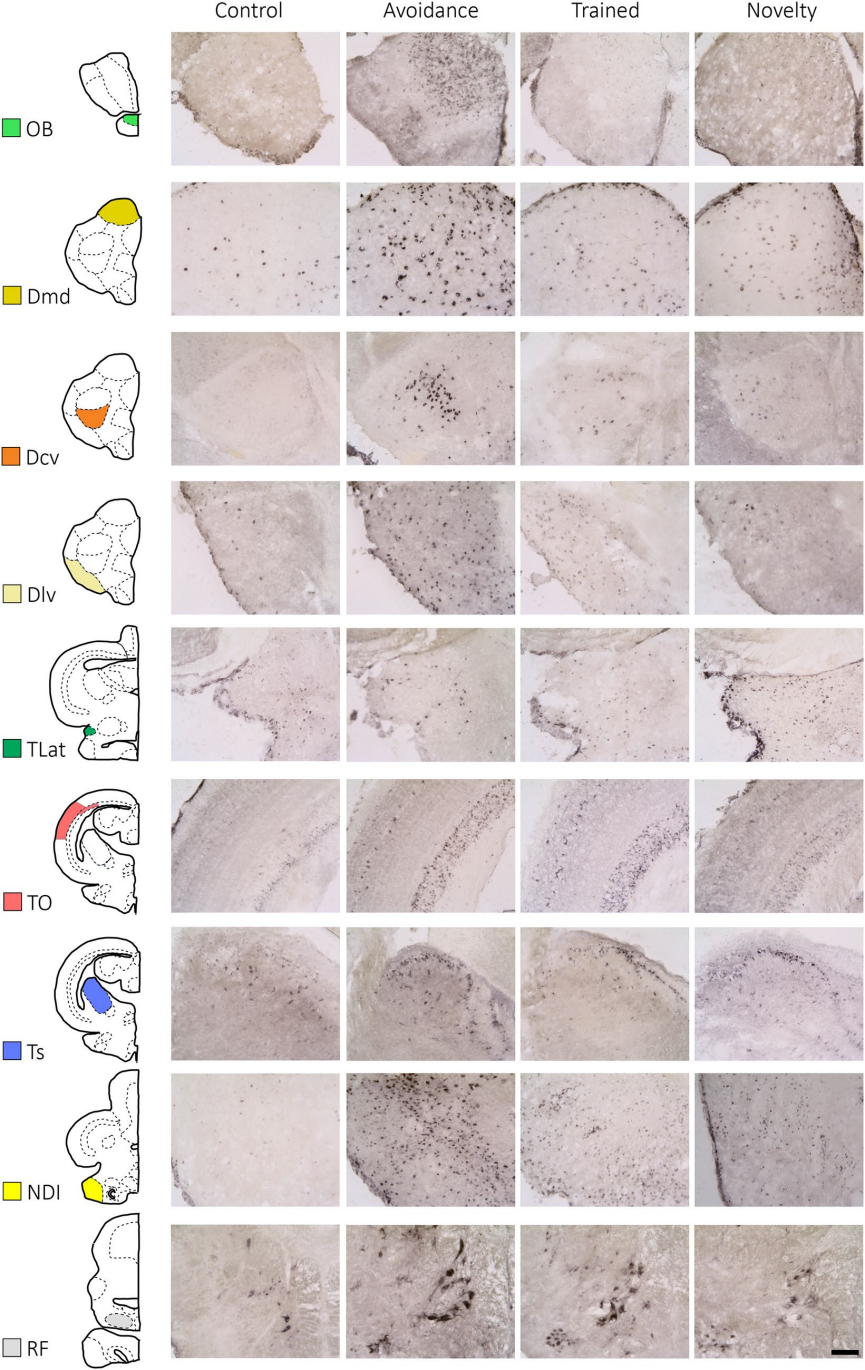
Staining for pS6 in 9 different brain areas (OB, Dmd, Dcv, Dlv, TLat, TO, TS, IL, and RF) in the four groups. Scale bar on the bottom right: 100 μm for each picture

**Fig. 4:**
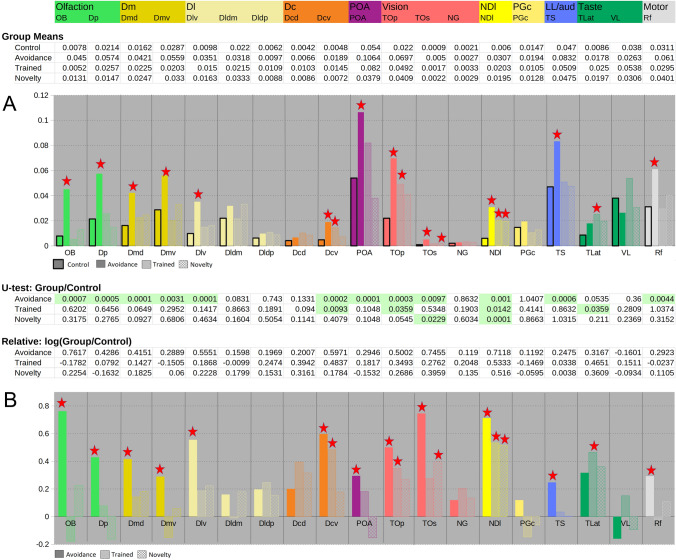
Results of brain areas analysis. Color code refers to Figs. 2 and 3. Graph A shows the mean pS6 staining intensity for all four groups in the 19 brain areas investigated. Graph B shows the values of the three learning groups relative to the control group (log ratios). The green highlighted values in the *U*-test table indicate *p* values below 0.05 and correspond to the asterisks in Graph B

After the analysis of the potentially induced behavioral components, pS6 staining was analyzed in 19 brain areas (see Table 1). Figure 3 shows examples of the staining in some selected areas in the four groups. For each group and brain region, the total area of stained cells was measured and divided by the total area selected. Then, we determined the activation in each brain area for each experimental group relative to the control group and log-transformed the data (Fig. 4). The stars indicate differences that are significant (*p* < 0.05) according to a *U*-test.

As anticipated, the avoidance group showed activation in many brain areas (see Figs. 3 and 4). The visual areas (TO and NDl) as well as the TS (lateral line/auditory centers) were activated. The reticular formation was also activated in this group. Several telencephalic areas were also positively stained, especially the olfactory-related OB and Dp. The avoidance group was also the only one that showed activation in the POA. In the other groups, fewer brain parts were activated. The only area that is activated in all learning groups is the nucleus diffusus of the inferior lobe.

## Discussion

The immediate early gene markers *c-fos* and *egr-1* and, more recently, the phosphorylation of the ribosomal protein S6, have been used as tools to study neuronal activation in diverse behavioral situations in fishes. Activation can occur at different levels, from sensory information processing to decision-making circuits to centers controlling emotional and hormonal states, premotor and motor areas. In fact, the first description of *c-fos* expression in fish was obtained by electrically stimulating motor neurons in the spinal cord and behaviorally by eliciting startle responses, which activate Mauthner cells and other reticular neurons in the rainbow trout (Bosch et al. 1995). The only other motor center that has been investigated with these activity markers in fish is the vocal motor nucleus in the sound-producing midshipman (Mohr et al. 2018; Schuppe et al. 2021). The majority of studies involving IEGs have focused on social behavior and decision-making circuits in the telencephalon and the diencephalon, e.g. the preoptic area, and the hypothalamus. The periaqueductal gray has also been investigated because of its role in emotional and motivational behaviors in mammals (Schuppe et al. 2021; Wood et al. 2011; Wong et al. 2012; Desjardins et al. 2010; Maruska et al. 2013), similar to the raphe superior (Desjardins et al. 2010; Wood et al. 2011; Butler et al. 2018). The only areas outside of the forebrain, that have been assessed in any detail are the tectum and the cerebellum.

In most studies, only a specific sensory modality and its relevant sensory pathways were investigated. For example, Butler and Maruska (2016) studied the role of the lateral line in social communication in cichlids and included in their analysis all major areas involved in the lateral line pathway, leading from the medulla to the diencephalon. Acoustic pathways were investigated in the midshipman (Mohr et al. 2018, Tripp et al. 2019, 2020). Finally, the gustatory system has been investigated in species that show bower or castle building during courtship (York et al. 2018, 2019). In most other studies on social behavior, the relevant sensory modality was not specified, and accordingly, sensory centers were not investigated (with the exception of the tectum opticum).

Few studies have focused on non-social behaviors. Lau et al. (2011) studied the innate avoidance of light, to describe the decision-making circuits. Messina et al. (2020, 2022) were investigating the habituation to a complex visual pattern in zebrafish, where the number of objects had to be remembered. Although they noted an upregulation of *c-fos* and *egr-1* in the tectum in response to object size (Messina et al. 2020), they focused on the telencephalon in a follow-up study (Messina et al. 2022), since they were more interested in the counting aspect of the task than in the size discrimination. Rodriguez-Santiago et al. (2021) investigated visual learning in a social context and found that the social aspect (but not the learning per se) was activating various telencephalic areas, but they did not investigate specific visual areas outside the telencephalon.

The aim of this project was to investigate some of the neural substrates involved in visual learning in the cichlid *Pseudotropheus zebra*. We excluded social contacts, but other behavioral components are certainly present to a variable degree and can hardly be excluded. Accordingly, we included in our analysis sensory areas, decision-making circuits in the telencephalon, the preoptic area, and the reticular formation, which is involved in locomotion. In contrast to many other studies, we were not focusing on one aspect of the behavioral task but tried to analyze and describe the entire behavioral situation with all of its components and correlate it with the activity in a large number of brain areas. Briefly summarized our results show the following:

1. The control group did not receive any sensory-motor or learning treatment. The fish were held in the tank for a week before being sacrificed. They were fed once a day, except the last day. This group shows baseline activity of pS6 in all areas. The activation of pS6 in the other groups (2–4) was measured relative to the baseline activity in the control group.
2. In the stress/avoidance group, fish were moved to a different tank and were chased with a net for one hour. Many sensory areas were activated. Olfactory stimulation correlated with the activation of OB and Dp, and was likely induced by the presence of new water and the net. The latter also seemed to have caused activation of the visual system, reflected by the strong activity of pS6 in both TOp and TOs. The lateral line/hearing system also showed activation (indicated by the activity of pS6 in TS) possibly due to the movement of the net in the water or noises made by the experimenter. There was also activity of pS6 in the preoptic area, which appears to be a stress-related response. In addition, the enforced locomotion activated the reticular formation, a premotor area. The avoidance learning component likely caused activation of the dorsomedial part of the telencephalon and the inferior lobes.
3. In the active training group, fish were trained every day to choose the correct target to get food. Once the fish reached the learning criterion (≥ 70% correct choices three times in a row), it was sacrificed. Compared to the control group, there was a little additional signal, possibly due to the continuous repetition of training. However, there was an increase in pS6 activity in the torus lateralis, a brain area associated with a taste that was likely activated by receiving a food reward. In addition, the inferior lobes together with both layers of the tectum opticum (TOp and TOs) were activated—possibly due to visual learning.
4. In the habituation/novelty group, staining was also similar to the control group. During the habituation phase, the stimulus was shown to the fish for 5 days, followed by a food release. On the last day, the novelty stimulus was shown. The presentation of a novel stimulus appears to be associated with a small increase in the activity of pS6 in the tectum (vision) and the inferior lobes.

The changes in activity in the different brain areas correlate with the different behavioral responses observed in the four groups. Common to all experimental groups is a strong activation of the inferior lobes. This may be explained by the fact that a visual learning component is present in all groups, except the control group. Higher cognitive functions are often thought to be located in the telencephalon, as is the case in mammals. However, lesion studies and further anatomical evidence have shown that the telencephalon of teleosts is less important for many sensory, motoric and cognitive tasks. Lesion studies have shown that the lesion/ablation of the telencephalon in teleosts has little effect on many behaviors (for a review see Calvo and Schluessel 2021), in particular, basic behaviors—such as swimming, feeding, and reproduction—are not affected by lesions at all (Steiner 1888; Bethe 1899; Rizzolo 1929). However, more intricate behaviors such as the reproductive behavior in sticklebacks (Schonherr 1955) may be heavily impacted, e.g. male sticklebacks show severe deficits in place memory without a telencephalon (e.g. the male does not show the female the correct entrance of the nest or does not find the nest itself). Similar results were found for Tilapia (Aronson 1948). A few studies investigating the role of the fish telencephalon in learning demonstrated impairments in avoidance behavior (e.g. Flood et al. 1976; Davis and Kassel 1983; Overmier and Hollis 1983). Other learning experiments showed no involvement of the telencephalon in simple conditioning and object recognition tasks (Froloff 1925, 1928; Bull 1928; Nolte 1932), pointing to other non-telencephalic areas that may be responsible for the processing of such information.

In 1996, Salas et al. (1996a, b) stimulated new interest in investigating telencephalic functions by discovering that allocentric place memory is located in the telencephalon, which was confirmed in a number of studies. Subsequent studies found the place memory to be restricted to lateral parts of the telencephalon (see Rodríguez et al. 2021 for a review). Further studies showed that the medial parts of the telencephalon play an important part for avoidance learning similar to the amygdala of mammals (see Broglio et al. 2005). Although these studies have shown that some functions of the telencephalon may be conserved across vertebrates, there are still some important differences in the organization of sensory pathways.

Anatomical studies on visual pathways in fish suggest that the major target of retinal fibers is the tectum in the midbrain (Northcutt and Wullimann 1988; Nieuwenhyus et al. 1998). There appears to be no prominent direct thalamic relay of retinal information to the telencephalon. The telencephalon receives visual and other sensory information, but through indirect routes via the preglomerular nuclei, which are part of a posterior tuberal area that is highly derived and elaborated in teleosts (Nieuwenhyus et al. 1998; Rodríguez et al. 2021). These ascending projections may serve the special functions that reside in the telencephalon like allocentric place memory and emotional learning, but skills such as general object recognition and egocentric spatial memory are probably organized in other di-and mesencephalic areas (Rodríguez et al. 2021). Several accessory areas are reciprocally connected with the tectum like the nucleus isthmi (Xue et al. 2001; Northmore and Gallagher 2003), torus longitudinalis (Wullimann 1994; Xue et al. 2003), and the pretectal areas (Fernald and Shelton 1985; Striedter and Northcutt 1989). A more complex pathway is reaching the inferior lobes via the nucleus corticalis and nucleus glomerulosus (Wullimann and Meyer 1990; Butler et al. 1991; Shimizu et al. 1999; Ahrens and Wullimann 2002; Yang et al. 2007). This system is especially prominent in spiny ray-finned fishes (acanthopterygian). The inferior lobes are located lateral to the traditional hypothalamus, a structure that is shared by all vertebrates. The inferior lobes, in contrast, are present only in teleosts and not found in any other vertebrate group. A recent study suggests that the inferior lobes are not derived from the forebrain like the hypothalamus, but are of mesencephalic origin (Bloch et al. 2019). These anatomical data indicate that the inferior lobes are involved in functions different from the ‘traditional’ hypothalamus. Our study showed for the first time that the inferior lobes, particularly the nucleus diffusus, are activated in all three visual learning situations. This is the first physiological evidence of the role of this structure in visual discrimination and memory formation, which is a common component of the behavior experienced in all treatment groups.

## Conclusion

The activation of ribosomal proteins can be detected in many brain areas and corresponds well with specific behavioral responses present in the four different control and learning situations investigated in this study. The only area consistently activated in all three treatment groups was the nucleus diffusus. It is located in the inferior lobes and the target of a prominent visual pathway originating in the tectum via the nucleus corticalis and the nucleus glomerulosus. Our study shows for the first time that this pathway may be involved in visual object recognition and memory formation. The inferior lobes may thus be one of the most important structures for higher cognitive functions outside of the telencephalon.

## Supplementary Information

Below is the link to the electronic supplementary material.

Supplementary file1 (PDF 2404 KB)

## Data Availability

The datasets generated during and/or analyzed during the current study are available from the corresponding author upon reasonable request.

## Abbreviations

*A*: Anterior thalamic nucleus
*ATN*: Anterior tuberal nucleus
*CC*: Crista cerebellaris
*CM*: Corpus mammilare
*Cor*: Corpus cerebelli
*CP*: Central posterior thalamic nucleus
*Dc*: Central division of the dorsal telencephalon
*Dcd*: Dorsal subdivision of the central division of the dorsal telencephalon
*Dcv*: Ventral subdivision of the central division of the dorsal telencephalon
*Dd*: Dorsal division of the dorsal telencephalon
*Dl*: Lateral division of the dorsal telencephalon
*Dld*: Dorsal subdivision of the lateral division of the dorsal telencephalon
*Dlv*: Ventral subdivision of the lateral division of the dorsal telencephalon
*Dm*: Medial division of the dorsal telencephalon
*Dmd*: Dorsal subdivision of the medial division of the dorsal telencephalon
*Dmv*: Ventral subdivision of the medial division of the dorsal telencephalon
*DON*: Descending octaval nucleus
*Dp*: Posterior division of the dorsal telencephalon
*DP*: Dorsal posterior thalamic nucleus
*E*: Entopeduncular nucleus
*EG*: Eminentia granularis
*fr*: Fasciculus retroflexus
*G*: Granular layer of the cerebellum
*Gl*: Granular layer of the olfactory bulb
*Hab*: Habenula
*IL*: Inferior lobe
*ILc*: Inferior lobe, central nucleus
*ILrec*: Inferior lobe, nucleus of the lateral recess
*IP*: Nucleus interpeduncularis
*ll*: Lateral lemniscus
*M*: Molecular layer of the cerebellum
*Ma*: Mauthner cell
*Mg*: Magnocellular octaval nucleus
*ME*: Median eminence of the hypothalamus
*mlf*: Medial longitudinal fascicle
*mIII*: Oculomotor nucleus
*MON*: Medial octavolateral nucleus
*mV*: Trigeminal motor nucleus
*mX*: Vagal motor nucleus
*ND*: Nucleus diffuses
*NDl*: Nucleus diffusus pars lateralis
*NDm*: Nucleus diffusus pars medialis
*NG*: Nucleus glomerulosus
*NGT*: Tertiary gustatory nucleus
*NI*: Nucleus isthmi
*NLT*: Nucleus lateralis tuberis
*nlVal*: Nucleus lateralis valvulae
*NSC*: Suprachiasmatic nucleus
*OB*: Olfactory bulb/granular layer
*OT*: Optic tract
*PGc*: Commissural preglomerular nucleus
*PGm*: Medial preglomerular nucleus
*POA*: Preoptic area
*PTc*: Pretectal area, centralis
*PTco*: Pretectal area, corticalis
*PTpo*: Pretectal area, nucleus of the posterior commissure
*PTsm*: Pretectal area, superficialis magnocellularis
*PTsp*: Pretectal area, superficialis parvocellularis
*pV*: Principal trigeminal nucleus
*Ras*: Raphe superior
*RFi*: Inferior reticular formation
*RFm*: Medial reticular formation
*RFs*: Superior reticular formation
*SPV*: Stratum periventriculare of the tectum
*sVII*: Sensory root of the facial nerve
*TL*: Torus longitudinalis
*TLat*: Torus lateralis
*TO*: Tectum opticum
*TOp*: Periventricular layer of the tectum opticum
*TOs*: Superficial layer of the tectum opticum
*TS*: Torus semicircularis
*Val*: Valvula cerebelli
*Vd*: Dorsal nucleus of the ventral division of the telencephalon
*VIII*: Octaval nerve
*Vl*: Lateral nucleus of the ventral division of the telencephalon
*VL*: Vagal lobe
*VM*: Ventromedial thalamic nucleus
*Vv*: Ventral nucleus of the ventral division of the telencephalon
*X*: Vagal nerve
