# Supplementary material for: Brain areas activated during visual learning in the cichlid fish Pseudotropheus zebra
Source: Brain Struct Funct. 2023 Mar 15;228(3-4):859–73. doi: 10.1007/s00429-023-02627-w (PMC10147796; doi:10.1007/s00429-023-02627-w)
Supplement: Supplementary file 1 — Supplementary file1 (PDF 2404 KB) [file 429_2023_2627_MOESM1_ESM.pdf]

Supplementary Methods

Brain areas activated during visual learning in the cichlid fish *Pseudotropheus zebra*

R. Calvo, M. H. Hofmann, V. Schluessel

Corresponding author: R. Calvo, rcalvo@uni-bonn.de

Brain Structure and Function

Slide scanning
All slides were scanned with a Zeiss microscope equipped with a scanning table at a resolution of 1.6 um. Individual sections were extracted manually from the slide images and an image stack created for each individual brain. A computer program was made and used to handle the image stacks.

Identification of the areas
Sections were stained with diaminobenzidine (DAB) and no counter stain was used to allow automatic analysis. However, since the detection of the antibodies was made with a ABC-kit (Vectastain PK6100 elite), some background stain was visible possibly due to endogenous biotin. This allowed the identification of the areas even if no stained cells were present. Supplementary fig. 1 shows examples of all areas in a control fish with minimal basline pS6 stain and in the avoidance group with a high level of activation in many cell groups. The background stain caused by he ABC kit was also compared to series where a cresyl violet stain was applied to adjacent sections.
The areas choosen to count were all areas where a uniform distribution of pS6-labeled cells were present. It was not necessary to mark the entire area so the exact boundaries of the areas were not so important. We rather choose a subarea that was free of artefacts and also avoided the borders to other areas and ventricular and brain surfaces to exclude ependymal layers. Cell measurements were relative to the area defined and not to the total areas of the brain part.

Segmentation of the areas
The segmentation of the areas for the activity measurements was done by hand. The user selects a brain part to be segmented and the program randomly chooses an image stack from any of the four groups. The stack was presented by the program without any information about the individual or group selected. This assures that the user does not know which group was selected and can perform the segmentation blind. The user then identifies the brain part and defines an area to be measured. For each brain part, the user usually selected several sections and was using both sides of the brain to define as many areas as possible for a given brain part. There are four different experimental groups with 10 individuals each. 19 brain parts were measured in each individual and the total number of areas segmented was over 3000. Table 1 shows the number of areas defined for all individuals and all brain parts. To process all these areas, an automatic procedure was developed.

Image processing and activation measurement
After all areas were defined, the program extracted all subimages from the image stacks. The next step was to convert all to 8-bit gray scale and to equalize the histograms. Fig. 2A shows one section with the areas defined. Fig. 2B shows one extracted subimage with the polygon defining the area. The histogram below Fig. 2B shows the histogram with a peak at about 180. The image was then blurred and the blurred image subtracted from the original and 128 added. This resulted in an image with a peak histogram value of always 128, ensuring that all images have the same brightness (Fig. 2C). At the same time, the images were 'flattened' i.e. uneven background gradients were removed. This is important for the next step. An object detection algorithm searched for darker objects with a threshold of 114 and generated a chain code for each object (Fig. 2D). The area of each object was calculated from the chain code and all objects smaller than 5 square pixels discarded. The areas of the remaining objects was summed up and divided by the total segmented area. This gives the fraction of the area stained. The reason for calculating the area rather than counting the cells is that in the 30 µm sections, cells were sometimes so close together that they were detected as one object. If more cells were clustered the actual count would go down, but the area still would increase. So we calculated the fraction of the stained area and finally all areas belonging to the same brain part were averaged. The results are shown in Table 2.

Final adjustment of the variability and averaging within groups
Before averaging the values within groups for each brain part, another equalizing step was done. Some individuals showed consistently lower values for all brain parts than others. This increases the variability within groups and the detection of differences between groups is less sensitive. To remove this variability, the values for each individual was averaged over all brain parts and the original part values divided by the average. This would not only remove all staining differences within a group, but also all between groups. To restore the between group differences, a grant average was calculated for all individuals within a group and all brain parts and the individual means multiplied by the total group mean. With this procedure, the variability within groups was reduced without reducing at the same time the variability between groups.
After this final adjustment, means were calculated for each brain part and group. The results are shown in the figure 4 of the main publication.

Control: Randomizing group assignments.
Several tests were made to adjust the two critical parameters in the image processing. The first one is the subtraction of the original image from a blurred version. Too little blurring did not equalize the background good enough and too much did not completely eliminate background gradients. This could be clearly seen because stained cells were not detected correctly. The same holds true for the threshold to detect the cells. It has to be adjusted in a number of different areas so that all visible cells were detected correctly. After these adjustments, all areas were run with the same parameters.
To check for possible bias in the procedure, we run the analysis with the correct groups and found differences in the activation of the brain parts as shown in figure 3A. This is identical to the figure 4 in the main publication. Then we randomized the group assignments of the individuals so that each individual was assigned randomly to one of the four groups. As expected, this eliminates all differences between groups (Fig. 3B). This means the correct group assignment is important. The procedure is successful in showing differences in the activation pattern of the brain part, but only if the groups are sorted out correctly. That means that the differences in activation pattern are indeed due to the different treatments.

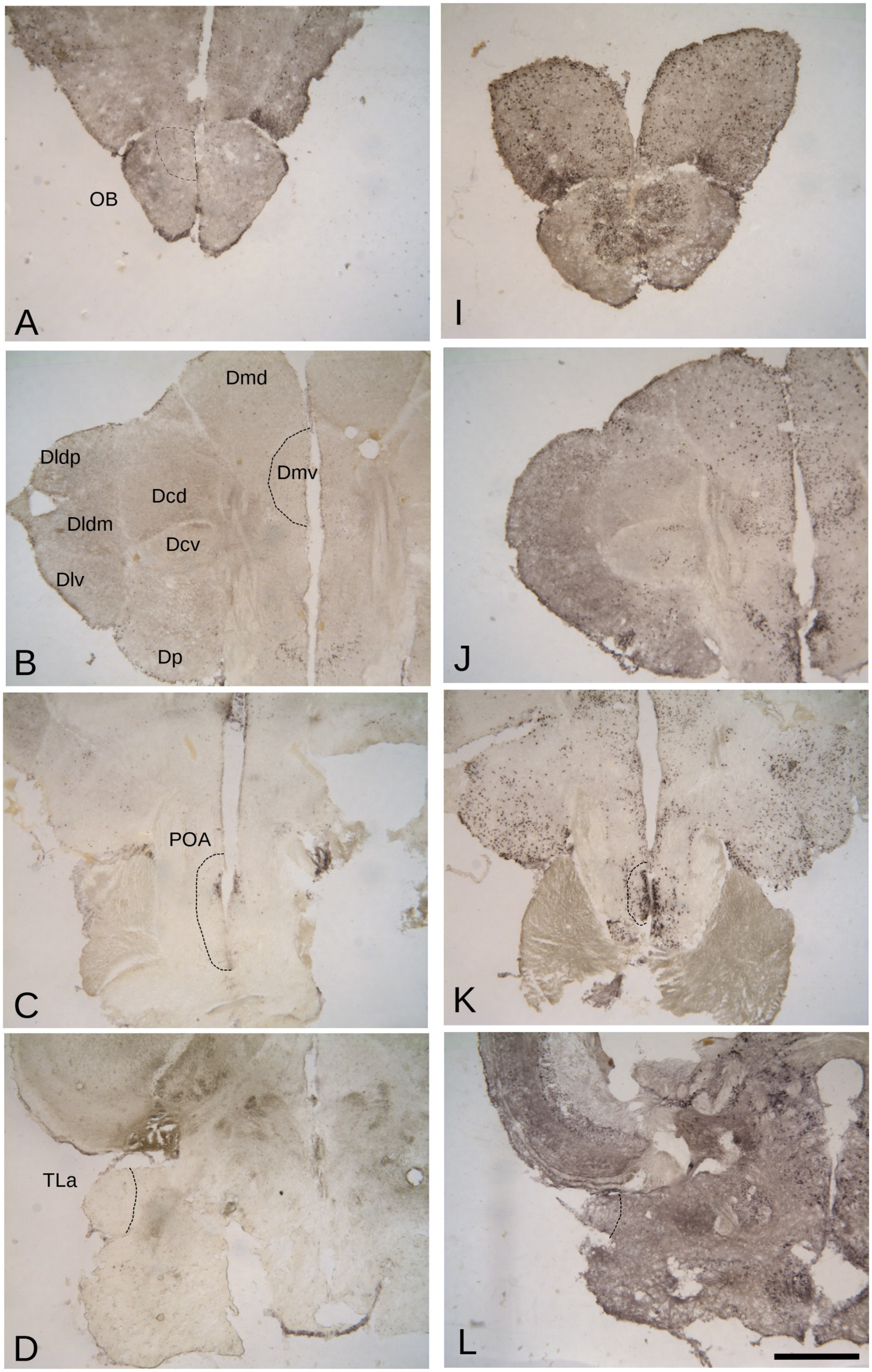

Fig. 1: Cross sections through the brains of a control fish (A-H) and a fish from the avoidance group (I-P) showing ps6-stained neurons and the background stain caused by the ABC-kit. Identification of all areas was possible even in the control group that showed minimal basline ps6 staining. For abbreviatins see Table1 of the main paper. Scale bar in L equals 500 μm.

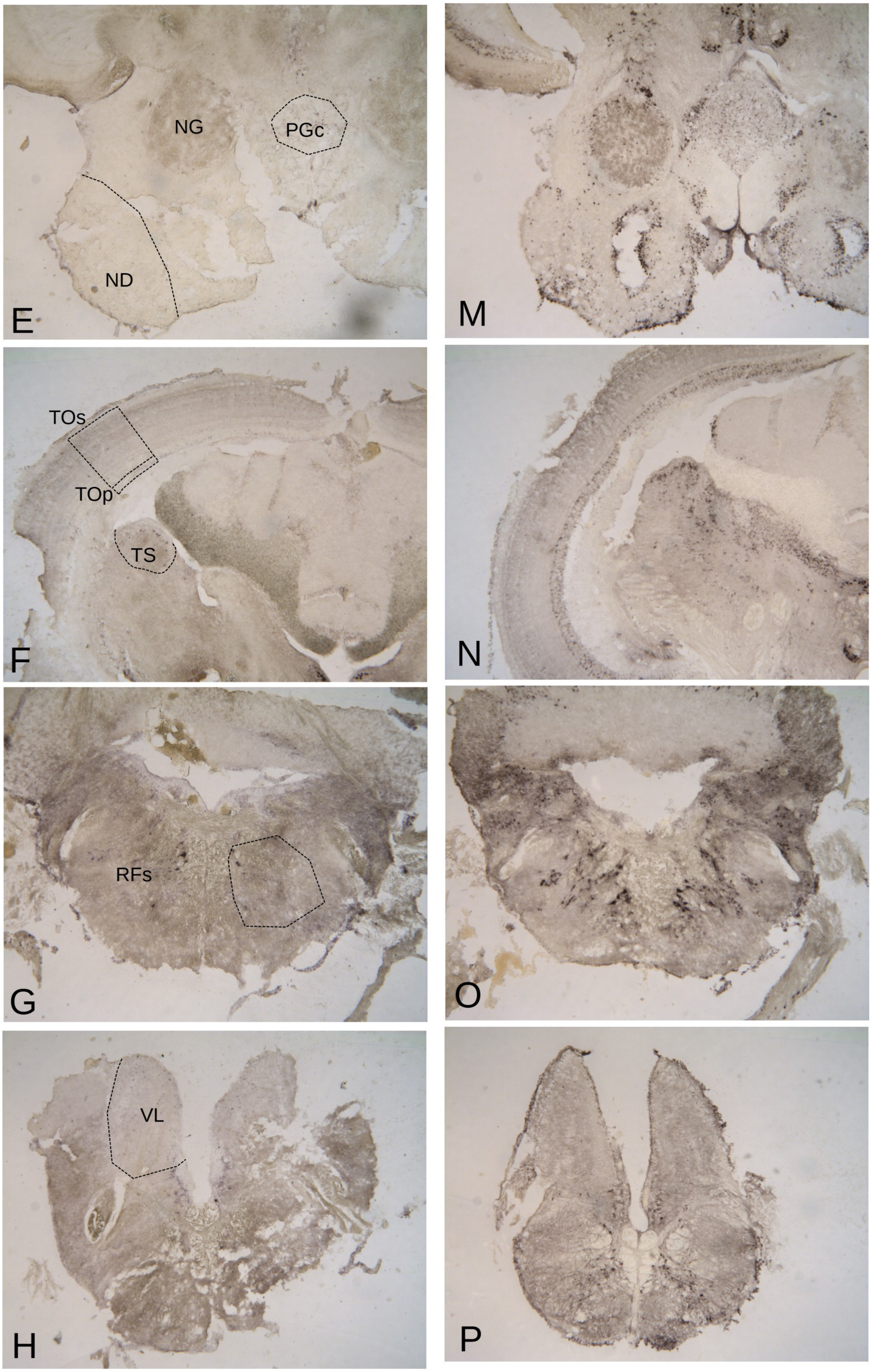

Fig. 1 continued

Supplementary Table 1 showing the number of areas per brain part in each individual of the four groups. Note that some brain parts could not be segmented in some individuals due to damaged brain parts or artifacts in the staining procedures.

Specimen	OB	Dp	Dm	Dmv	Dlv	Dldm	Dldp	Dcd	Dcv	POA	TOp	TOs	NG	NDI	NPT	TS	TLat	VL	Rf
1.CI	32	33	54	37	39	23	23	30	32	15	10	10	38	48	16	34	26	36	121
01	5	2	7	4	4	3	3	2	2	2	1	1	1	3	1	2	1	2	9
02	2	3	4	3		1	1			3	1	1	4	3	1	4	4		
03		2	2		3	3	3	3	4	1	1	1	5	4	1	6	2	5	20
04	10	4	4	4	5	2	2	4	4	1	1	1	6	5	3	1	3	3	7
05	5	7	11	5	5	2	2	3	4	1	1	1	7	6	1	3	1	4	10
06	4	4	7	6	5	3	2	3	3	2	1	1	2	6	1	3	2	4	10
07	3	7	3	5	5	3	4	6	6	2	1	1	6	7	2	5	4	4	20
08		1	6	6	6	3	3	3	3	1	1	1	2	4	1	4	3	6	16
09											1	1	2	4	3	3	4	6	16
10	3	3	10	4	6	3	3	6	6	2	1	1	3	6	2	3	2	2	13
2.ST	43	43	80	55	47	28	31	43	47	21	10	10	23	41	13	60	30	38	107
01	1	3	13	7	3	3	3	4	5	2	1	1	3	4	1	7	4	2	10
02	4	5	7	3	6	4	4	4	4	2	1	1	2	7	1	10	1	4	8
03	6	2	6	1	3	4	3	8	9	3	1	1		3		3	3	4	11
04	9	3	4	4	4	3	3	4	4	2	1	1	4	5	2	6	2	2	12
05	4	5	8	6	6	4	4	4	4	2	1	1	3	4	2	4	8	2	12
06	5	5	12	8	6	2	3	3	3	2	1	1	2	5	3	9	2	4	10
07	4	5	10	8	6	2	5	4	4	3	1	1	5	3	1	4	4	6	12
08	2	5	7	5	3	2	3	2	4	1	1	1	1	3		6	1	7	15
09	6	5	10	10	7	2	2	5	5	2	1	1	2	4	1	7	3	6	7
10	2	5	3	3	3	2	1	5	5	2	1	1	1	3	2	4	2	1	10
3.TR	38	41	39	20	40	13	15	29	30	19	10	10	46	46	15	44	27	35	108
01	1	4	3		3	1	1	3	4		1	1	1	2		2		6	13
02	6	7	3	2	8	3	3	4	4	3	1	1	5	8	3	6	2		18
03		4	7	4	4	2	2	4	4	1	1	1	5	1	1	5	2	5	12
04			2	2	3	2	2	2	2	1	1	1	5	4	2	3	3	2	9
05	10	5	3	3	6	2	4	2	2	3	1	1	6	7	1	3	2	6	12
06	2	5			3	2	2			3	1	1	4	4	2	4	3	5	9
07	6	2	8	4	4			4	4	2	1	1	3	3		3	3		7
08	3	6	4		2			4	4	1	1	1	8	10	2	6	2	4	17
09	4	2	4		4			4	4	2	1	1	6	4	4	6	4	1	
10	6	6	5	5	3	1	1	2	2	3	1	1	3	3		6	6	6	11
4.ME	43	38	43	35	39	26	26	31	32	17	11	11	50	42	18	44	22	36	105
01		7	12	8	5	3	3	4	4	3	1	1	8	6	2	2	4	4	15
02										2	1	1	5	6	2	5	4	6	7
03	2	4	9	4	4	4	4	2	2	3	1	1	7	4	3	12	3	4	12
04	8	5	4	3	5	4	4	9	9	2	2	2	11	3	3	7	2	4	16
05	5	4	4	4				4	4	2	1	1	5	5	2	5	1	2	6
06	4	4	3	3	5	3	3	4	5	2	1	1	3	4	1	1	1	2	8
07	13	2	3	4	4	3	3	3	3		1	1	2	3		2	2	2	9
08	9	4	3	3	5	2	2	2	2	2	1	1	3	4	2	1	1	6	11
09		5	3	3	5	3	3	2	2		1	1	4	3	3	4	2	2	8
10	2	3	2	3	6	4	4	1	1	1	1	1	2	4		5	2	4	13

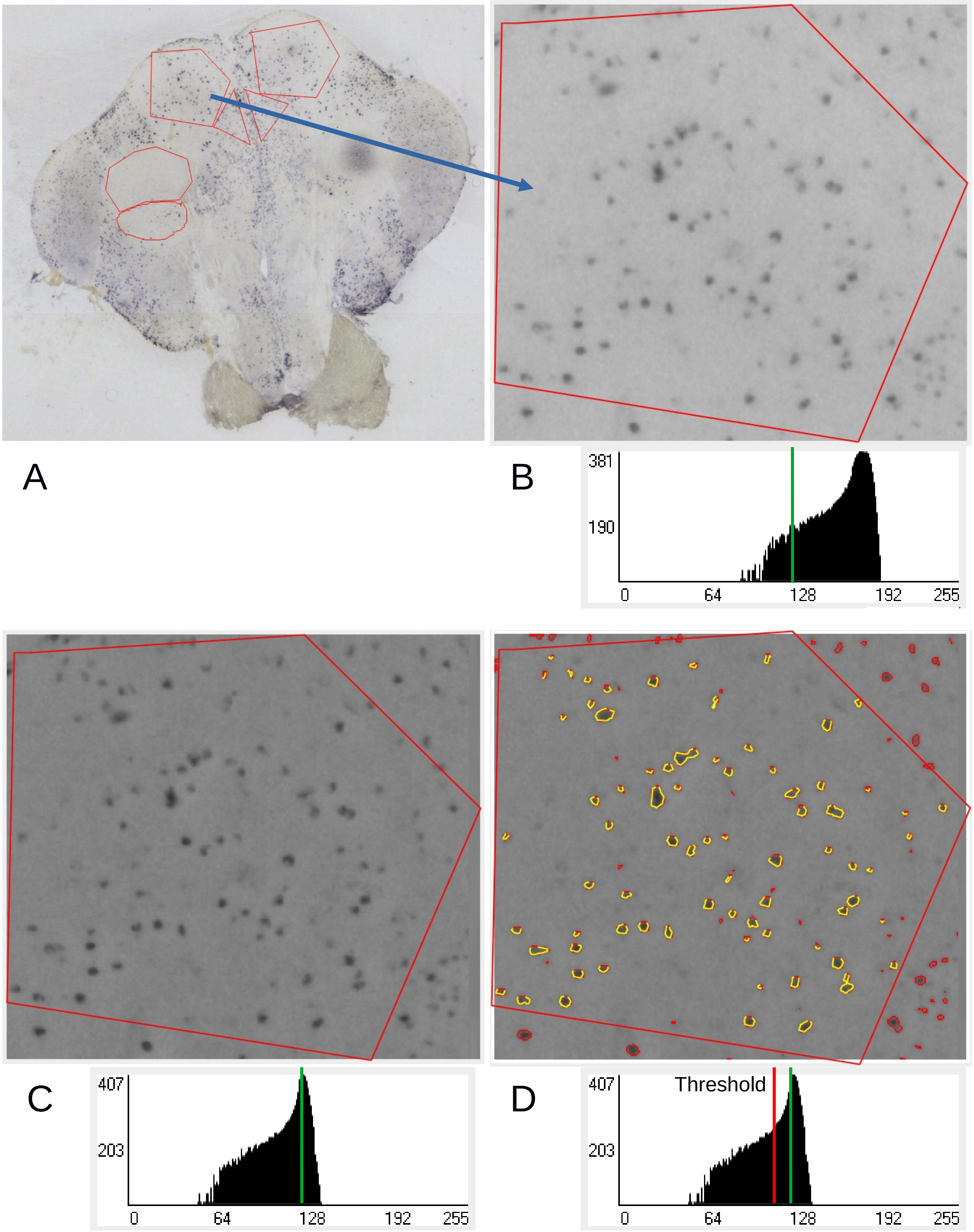

Supplementary Figure 2. Steps in the image analysis of the areas. A) Segmentation. B) Extraction of individual areas. C) Histogram equalization and D) Detection of stained cells

Supplementary Table 2 showing the fraction of the stained area relative to the total area segmented for each brain part and individual.

Specimen	OB	Dp	Dm	Dmv	Dlv	Dldm	Dldp	Dcd	Dcv	POA	TOp	TOs	NG	NDI	NPT	TS	TLat	VL	Rf
1.CI	0.0112	0.0174	0.01	0.0186	0.0078	0.0189	0.0055	0.0044	0.0048	0.0412	0.0187	0.0008	0.0025	0.0048	0.0119	0.0378	0.0112	0.03	0.0252
01	0.003	0.0318	0.009	0.0154	0.0021	0.011	0.0015	0.0009	0.001	0.0395	0.0091	0.0001	0.0006	0.0047	0.0099	0.0414	0.0037	0.0383	0.0109
02	0.003	0.041	0.033	0.0362		0.0289	0.0231			0.06	0.0539	0.0026	0.0077	0.005	0.006	0.0944	0.016		
03		0.0335	0.0494		0.017	0.032	0.0133	0.0226	0.028	0.0982	0.0731	0.002	0.0094	0.0202	0.0609	0.0653	0.0494	0.0761	0.071
04	0.0195	0.0401	0.0211	0.0466	0.0293	0.0639	0.019	0.0158	0.0099	0.0564	0.0374	0.002	0.0026	0.0154	0.0624	0.1235	0.0185	0.0177	0.0181
05	0.0126	0.0083	0.0101	0.0265	0.0073	0.0076	0.0002	0.0014	0.0028	0.0533	0.0035	0.0012	0.0004	0.0024	0.002	0.0391	0.0002	0.025	0.0375
06	0.0083	0.0108	0.0077	0.0067	0.0041	0.0108	0.0028	0.0007	0.0021	0.0237	0.0085		0.0002	0.0025		0.0272	0.0034	0.0476	0.0091
07	0.0006	0.01	0.0113	0.0255	0.0078	0.0079	0.0047	0.002	0.0011	0.0372	0.0238	0.0002	0.0019	0.0021	0.0041	0.023	0.0021	0.0165	0.0153
08		0.0000	0.003	0.0041	0.0013	0.0066		0.0003	0.0001				0.0005	0.0051		0.0047	0.0035	0.0277	0.0118
09											0.0068		0.0008	0.0024	0.0028	0.021	0.0055	0.0199	0.0188
10	0.001	0.0038	0.0046	0.0113	0.0032	0.0161	0.0008	0.0008	0.0015	0.0254		0.0001	0.0006	0.0032	0.0074	0.006	0.0031	0.0052	0.0198
2.ST	0.0467	0.0497	0.0365	0.0487	0.031	0.0338	0.0117	0.0073	0.0192	0.1026	0.0811	0.0056	0.0041	0.0338	0.0145	0.0743	0.02	0.0382	0.0621
01	0.1196	0.0543	0.036	0.053	0.0386	0.042	0.0049	0.0055	0.0157	0.0924	0.0845	0.011	0.0093	0.0177	0.0618	0.0948	0.0436	0.021	0.0785
02	0.0121	0.0763	0.0485	0.0689	0.0426	0.0769	0.0296	0.0132	0.0205	0.1146	0.0933	0.0058	0.0004	0.0496	0.0478	0.0831	0.0333	0.0404	0.0809
03	0.0782	0.0795	0.0445	0.0534	0.0402	0.0392	0.0315	0.0076	0.0211	0.132	0.1691	0.0094		0.0787		0.081	0.0351	0.0695	0.1263
04	0.0496	0.0409	0.0429	0.0517	0.032	0.026	0.0025	0.004	0.0178	0.1293	0.1083	0.0118	0.0047	0.0441	0.0457	0.0776	0.0042	0.0099	0.0553
05	0.017	0.0312	0.0164	0.0242	0.0121	0.006		0.0014	0.0039	0.0459	0.0399	0.0035	0.0000	0.0015	0.0006	0.0661	0.0095	0.0011	0.0102
06	0.0083	0.024	0.0213	0.0275	0.0125	0.0069	0.0001	0.0003	0.0043	0.0338	0.0061	0.0002	0.0002	0.0045	0.0007	0.0221	0.0034	0.0081	0.0169
07	0.0476	0.0547	0.0538	0.0559	0.0523	0.0227	0.0256	0.006	0.0268	0.1071	0.1114	0.002	0.007	0.0326	0.005	0.0879	0.036	0.0831	0.0937
08	0.0555	0.0622	0.04	0.0482	0.0386	0.0362	0.0148	0.0092	0.0321	0.1258	0.0515	0.0063	0.0019	0.0374		0.1129	0.0048	0.0172	0.0603
09	0.0581	0.0552	0.0398	0.0505	0.0301	0.0574	0.0019	0.01	0.0184	0.1129	0.0488	0.0015	0.0004	0.0376	0.0038	0.0643	0.009	0.0369	0.077
10	0.022	0.051	0.0316	0.0657	0.0389	0.02	0.0023	0.0121	0.0286	0.1178	0.0357	0.0014	0.003	0.0361	0.008	0.0711	0.0106	0.0075	0.0461
3.TR	0.003	0.0235	0.0191	0.0189	0.0122	0.0172	0.0097	0.0103	0.0148	0.0472	0.0449	0.0016	0.0029	0.0127	0.0051	0.031	0.0133	0.0611	0.0319
01	0.0399	0.069	0.0562		0.0612	0.0479	0.0259	0.0587	0.0562		0.0738	0.0061	0.0043	0.0179		0.1128		0.1051	0.0724
02	0.0012	0.0251	0.0143	0.004	0.0071	0.0091	0.0003	0.0009	0.0029	0.0316	0.0013	0.0001	0.0028	0.0181	0.0006	0.0156	0.0268		0.0149
03		0.0376	0.0248	0.0235	0.0167	0.0069	0.0049	0.0125	0.0174	0.0862	0.1017	0.0055	0.012	0.0655	0.0449	0.0842	0.071	0.0455	0.032
04			0.0139	0.0155	0.0146	0.0086	0.0167	0.0127	0.0282	0.05	0.028	0.0001	0.0042	0.0215	0.0081	0.0577	0.0161	0.0322	0.0288
05	0.0049	0.0264	0.0311	0.0409	0.0222	0.0422	0.0175	0.0131	0.0135	0.0611	0.1187	0.004	0.0029	0.0228	0.0034	0.072	0.0296	0.0306	0.0377
06	0.0006	0.0152			0.0185	0.0289	0.0098			0.0406	0.0699		0.0008	0.0096	0.0036	0.0358	0.0047	0.0371	0.0086
07	0.0005	0.0048	0.0041	0.0009	0.0006			0.0005	0.002	0.0681	0.0198	0.0003	0.0007	0.006		0.0299	0.0026		0.0033
08	0.0052	0.0133	0.0219		0.0016			0.0076	0.0071	0.0751	0.0331	0.0009	0.0012	0.0089	0.0145	0.0282	0.0116	0.1263	0.0457
09	0.0006	0.0002	0.0002		0.0004			0.0002	0.0001	0.0053	0.0004		0.0000	0.0001	0.0002		0.0003	0.0019	
10	0.0022	0.0123	0.0191	0.032	0.0111	0.0146	0.0105	0.007	0.0101	0.0765	0.0258		0.0012	0.0021		0.0208	0.0122	0.0447	0.0306
4.ME	0.0146	0.0135	0.0205	0.0315	0.014	0.0265	0.0088	0.0055	0.0053	0.038	0.0463	0.0022	0.0039	0.0214	0.0141	0.0566	0.0174	0.0338	0.0407
01		0.0186	0.0214	0.0334	0.0052	0.0181	0.003	0.0072	0.0084	0.043	0.0491	0.0009	0.0012	0.0213	0.0035	0.0039	0.039	0.0752	0.0729
02										0.0218	0.0649	0.0028	0.0068	0.0393	0.0185	0.0412	0.0046	0.0353	0.0579
03	0.0105	0.0285	0.0237	0.0276	0.0239	0.0369	0.029	0.0067	0.0237	0.0691	0.0945	0.0048	0.0076	0.0166	0.0318	0.0959	0.0191	0.0413	0.0779
04	0.0119	0.0163	0.0098	0.0266	0.0036	0.0037	0.0002	0.001	0.0003	0.0502	0.0162	0.0035	0.0054	0.0198	0.0058	0.0171	0.0085	0.0521	0.021
05	0.0237	0.0108	0.0174	0.042				0.0034	0.0025	0.0504	0.0082	0.0006	0.0013	0.0232	0.0347	0.0977	0.0577	0.0477	0.0564
06	0.0005	0.0098	0.0222	0.0135	0.0031	0.0285	0.0015	0.01	0.0064	0.0198	0.0041		0.0008	0.0178	0.0027	0.0605	0.0038	0.0042	0.0387
07	0.0283	0.0138	0.0325	0.0315	0.0278	0.0375	0.0141	0.0068	0.0065		0.0464	0.0019	0.0011	0.0128		0.0395	0.0019	0.0124	0.0426
08	0.0054	0.0053	0.0346	0.0429	0.0336	0.0171	0.0137	0.0058	0.0077	0.0229	0.0367	0.0015	0.0021	0.0131		0.0516	0.0138	0.012	0.0164
09		0.0057	0.013	0.0161	0.0165	0.0528	0.0102	0.0104	0.0087		0.0738	0.0028	0.0011	0.0142	0.0114	0.0379	0.0054	0.0234	0.0222
10	0.0091	0.0155	0.025	0.0392	0.009	0.0414	0.0028	0.0157	0.0024	0.0221	0.0292	0.001	0.0014	0.0102		0.0273	0.0448	0.0042	0.0095

	Olfaction		Dm		Dl			Dc		POA	Vision			NDI	PGc	LL/aud	Taste		Motor
	OB	Dp	Dmd	Dmv	Dlv	Dldm	Dldp	Dcd	Dcv	POA	TOp	TOs	NG	NDI	PGc	TS	TLat	VL	Rf
**Group Means**																			
Control	0.0078	0.0214	0.0162	0.0287	0.0098	0.022	0.0062	0.0042	0.0048	0.054	0.022	0.0009	0.0021	0.006	0.0147	0.047	0.0086	0.038	0.0311
Avoidance	0.045	0.0574	0.0421	0.0559	0.0351	0.0318	0.0097	0.0066	0.0189	0.1064	0.0697	0.005	0.0027	0.0307	0.0194	0.0832	0.0178	0.0263	0.061
Trained	0.0052	0.0257	0.0225	0.0203	0.015	0.0215	0.0109	0.0103	0.0145	0.082	0.0492	0.0017	0.0033	0.0203	0.0105	0.0509	0.025	0.0538	0.0295
Novelty	0.0131	0.0147	0.0247	0.033	0.0163	0.0333	0.0088	0.0086	0.0072	0.0379	0.0409	0.0022	0.0029	0.0195	0.0128	0.0475	0.0197	0.0306	0.0401

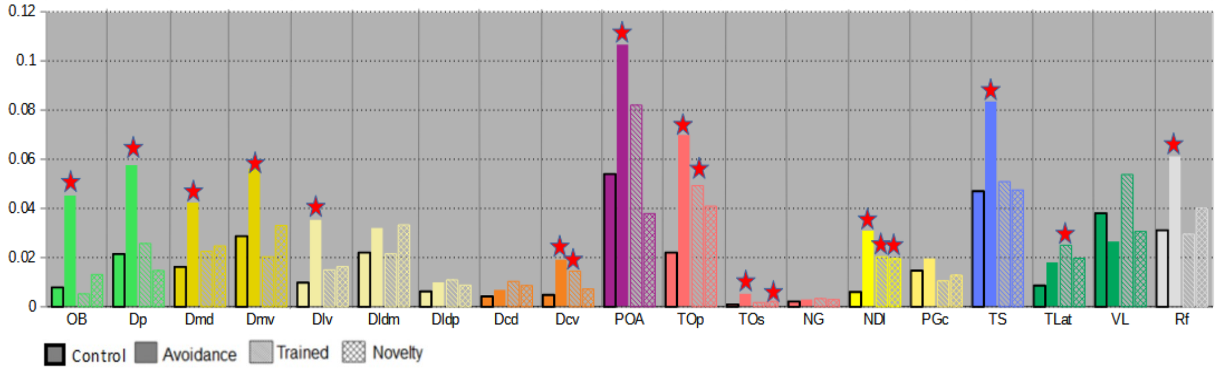

A

	Olfaction		Dm		Dl			Dc		POA	Vision			NDI	PGc	LL/aud	Taste		Motor
	OB	Dp	Dmd	Dmv	Dlv	Dldm	Dldp	Dcd	Dcv	POA	TOp	TOs	NG	NDI	PGc	TS	TLat	VL	Rf
**Group Means RANDOMIZED**																			
Control	0.0209	0.0312	0.027	0.037	0.0202	0.0274	0.0085	0.0075	0.0117	0.0727	0.0459	0.0026	0.0027	0.0194	0.0143	0.0567	0.0174	0.0352	0.0404
Avoidance	0.0202	0.0309	0.0269	0.0368	0.02	0.0272	0.0085	0.0075	0.0115	0.0723	0.0454	0.0026	0.0026	0.0191	0.0141	0.0567	0.0175	0.0355	0.0401
Trained	0.0206	0.0312	0.0271	0.0371	0.0201	0.0273	0.0084	0.0075	0.0117	0.0729	0.0457	0.0027	0.0027	0.0195	0.0143	0.0571	0.0174	0.0356	0.0405
Novelty	0.0211	0.031	0.0267	0.0365	0.0199	0.0273	0.0084	0.0074	0.0116	0.0724	0.0455	0.0027	0.0027	0.0194	0.0145	0.0567	0.0175	0.0355	0.0404

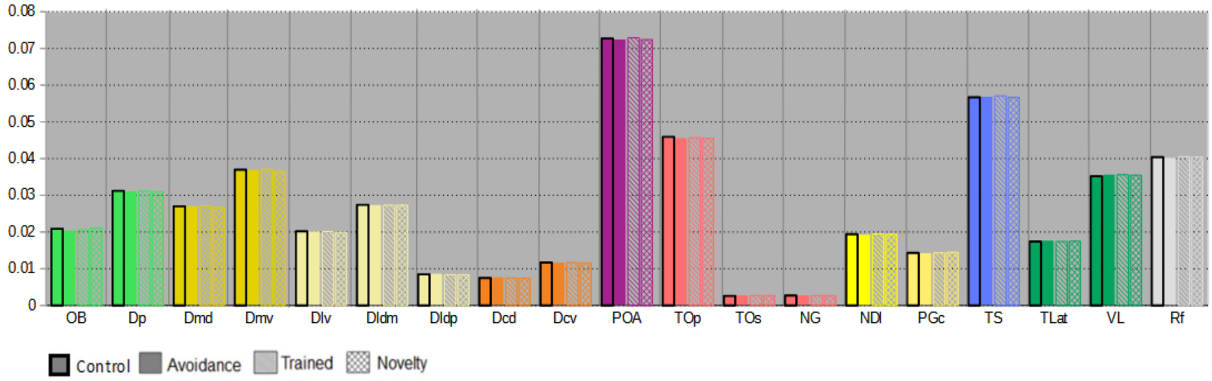

B

Supplementary Figure 3 showing in A) the within group averaged values with correct group assignments and in B) the results after randomizing the group assignments of the individuals. All differences between groups are eliminated if the individuals are not assigned to their correct treatment groups
